# Machine Learning-Enabled Medical Devices Authorized by the US Food and Drug Administration in 2024: Regulatory Characteristics, Predicate Lineage, and Transparency Reporting

**DOI:** 10.3390/biomedicines13123005

**Published:** 2025-12-08

**Authors:** Bassel Almarie, Luis Fernando Gonzalez-Gonzalez, Lucas Antônio dos Santos Barbosa, Amelie Lutz, Ulrich Grosse, Felipe Fregni

**Affiliations:** 1 Neuromodulation Center and Center for Clinical Research Learning, Spaulding Rehabilitation and Massachusetts General Hospital, Harvard Medical School, Boston, MA 02138, USA; 2 Graduate Program in Neuroscience, Federal University of Santa Catarina, Florianopolis 88040-900, SC, Brazil; 3 Department of Radiology, Stanford University School of Medicine, Stanford, CA 94305, USA; 4 Institute of Radiology, Cantonal Hospital Frauenfeld, 8500 Frauenfeld, Switzerland

**Keywords:** artificial intelligence, machine learning, medical device regulation, FDA 510(k), predetermined change control plans, algorithmic bias, health equity, demographic representation, regulatory transparency

## Abstract

**Background**: The US Food and Drug Administration (FDA) authorized over 690 machine learning (ML)-enabled medical devices between 1995 and 2023. In 2024, new guidance enabled the inclusion of Predetermined Change Control Plans (PCCPs), raising expectations for transparency, equity, and safety under the Good Machine Learning Practice (GMLP) framework. **Objective**: The objective was to assess regulatory pathways, predicate lineage, demographic transparency, performance reporting, and PCCP uptake among ML-enabled devices approved by the FDA in 2024. **Methods**: We conducted a cross-sectional analysis of all FDA-authorized ML-enabled devices in 2024. Data extracted from FDA summaries included regulatory pathway, predicate genealogy, performance metrics, demographic disclosures, PCCPs, and cybersecurity statements. Descriptive and nonparametric statistics were used. **Results**: The FDA authorized 168 ML-enabled Class II devices in 2024. Most (94.6%) were cleared via 510(k); 5.4% were cleared via De Novo. Radiology dominated (74.4%), followed by cardiovascular (6.5%) and neurology (6.0%). Non-US sponsors accounted for 57.7% of clearances. Among 159 510(k) devices, 97.5% cited an identifiable predicate; the median predicate age was 2.2 years (IQR 1.2–4.1), and 64.5% ML-enabled. Predicate reuse remained uncommon (9.9%). Median review time was 162 days (151 days for 510(k) vs. 372 days De Novo; *p* < 0.001). A total of 49 devices (29.2%) reported both sensitivity and specificity; 15.5% provided demographic data. PCCPs appeared in 16.7% of summaries, and cybersecurity considerations appeared in 54.2%. **Conclusions**: While 2024 marked a record year for ML-enabled device approvals and internationalization, uptake of PCCPs and transparent performance and demographic reporting remained limited. Policy efforts to standardize disclosures and strengthen post market oversight are critical for realizing the promises of GMLP.

## 1. Introduction

Artificial intelligence (AI) innovations have transitioned from experimental proofs of concept to regulated clinical products with remarkable speed. In the United States, one of the leading hubs for digital health innovation, the Food and Drug Administration (FDA) had authorized 692 AI/Machine Learning (ML)-enabled devices by 2023, representing a 20-fold increase compared to the mean annual approval rate between 1995 and 2015 [1]. This growth has sharpened long-standing questions about how to ensure the safety, effectiveness, and equity of technologies whose performance can evolve continuously in response to new data.

Regulatory bodies have begun to facilitate the marketing of these innovations while addressing the emerging challenges through a life-cycle, practice-oriented framework. In 2021, the FDA, Health Canada, and the United Kingdom Medicines & Healthcare products Regulatory Agency released ten “Good Machine Learning Practice” (GMLP) guiding principles, spanning rigorous software engineering, representative datasets, human-AI team performance, and post-deployment monitoring [2]. Building on GMLP principle 10, the FDA adapted guiding principles in early 2024 on Predetermined Change Control Plans (PCCPs)—a mechanism that enables sponsors to seek prospective authorization for specified algorithm updates, thereby aligning regulatory review with the rapid cadence of model iteration [3].

Yet technological innovation has outpaced regulatory frameworks, ensuring that AI devices are being developed and evaluated in ways that advance equity and transparency. A recent scoping review of 692 FDA-approved AI/ML devices between 1995 and 2023 found that only 3.6% reported race or ethnicity of validation cohorts, less than 1% provided socioeconomic information, and fewer than 2% linked to peer-reviewed performance studies. These gaps may exacerbate health disparities, particularly among vulnerable and underrepresented populations, and compromise the reliability of approved devices [4]. The consequences of inadequate demographic representation and algorithmic bias are not merely theoretical. For example, previous research demonstrated that a widely deployed population-health algorithm systematically underestimated illness severity in Black patients by relying on healthcare expenditures as a proxy for clinical need, thereby embedding structural inequities in healthcare access into clinical decision-making [5].

Regulatory documentation itself may further cloud the picture. One systematic review showed that nearly one-fifth of AI devices marketed in the United States described capabilities that were not reflected in their cleared indications for use, which raised concerns about “function creep” beyond the evidence base reviewed by the FDA [6]. Meanwhile, early uptake of PCCPs appears limited, and it remains unclear whether the new guidance has improved AI fairness disclosures, including performance metrics, demographic representativeness, or cybersecurity practices [7].

Recent analyses offer additional context for interpreting these gaps. Studies evaluating PCCPs highlight their potential to streamline oversight of evolving software while also identifying limitations in current regulatory readiness and stakeholder familiarity with these tools [8,9]. Complementary analyses in pediatric AI governance underscore ongoing risks related to insufficient demographic diversity and unclear accountability structures [10]. Broader assessments of U.S. AI/ML regulation likewise point to persistent gaps in data governance, monitoring rigor, and oversight of continuously learning systems, further underscoring why mechanisms such as GMLP and PCCPs have become central to regulatory modernization efforts [11].

The present study provides a comprehensive assessment of all AI/ML-enabled medical devices cleared or approved by the FDA during calendar year 2024. Focusing on regulatory pathways, predicate genealogies, approval timelines, reporting of performance and demographic data, cybersecurity provisions, and adoption of PCCPs, we aim to determine whether the most recent cohort of clearances signals substantive progress toward the goals articulated in GMLP or perpetuates previously documented shortcomings. Insights from this analysis are intended to inform ongoing policy deliberations and to guide clinicians, developers, and regulators seeking to balance rapid innovation with the imperatives of safety, effectiveness, and health equity.

## 2. Materials and Methods

### 2.1. Study Design and Data Source

We conducted a cross-sectional analysis of all ML-enabled medical devices approved by the FDA in 2024. Device data were extracted from the FDA’s official database of AI/ML-enabled medical devices (https://www.fda.gov/medical-devices/software-medical-device-samd/artificial-intelligence-and-machine-learning-aiml-enabled-medical-devices, accessed on 17 May 2025). As all materials are in the public domain, institutional review board oversight was not required.

### 2.2. Data Extraction

For each device, we systematically extracted device-level identifiers (submission number, manufacturer, sponsor country, specialty panel, intended use, and regulatory pathway), regulatory characteristics (device class, presence of PCCP, designation as software-as-a-medical-device [SaMD], cybersecurity statements, and pediatric indication), machine-learning characteristics (algorithm architecture where documented), bias and fairness variables (disclosure of demographic baseline variables including sex, age, and race/ethnicity), performance metrics (sensitivity, specificity, area under the receiver-operating-curve, positive/negative predictive value, and other task-appropriate metrics with confidence intervals where provided), and 510(k) genealogy data (submission numbers of primary and secondary predicates, reference devices, year of clearance, and ML-enablement status). Data were extracted manually from FDA 510(k) and De Novo decision summaries. Each variable was cross-checked for internal consistency across the device summary, labeling, and decision memo to ensure accuracy.

### 2.3. Regulatory Pathway and Genealogy Analysis

We categorized devices according to their regulatory pathway (510(k) or De Novo) and further classified 510(k) devices by submission type (Traditional, Special, or Abbreviated). For devices approved through the 510(k) pathway, we identified primary predicates and any additional predicates or reference devices cited in the substantial equivalence determination. We documented the approval date of each predicate to establish temporal relationships and calculated the time elapsed between predicate approval and subsequent device clearance.

To identify ML-enabled medical devices, we cross-referenced each predicate against the FDA’s official database of AI/ML-enabled medical devices. Each predicate was labeled as either ML-enabled or non-ML-enabled based on its inclusion in the FDA list. For each 510(k)-cleared device, we constructed a regulatory genealogy by identifying its primary predicate and determining whether that predicate was ML-enabled. We documented instances of predicate reuse and calculated the frequency with which specific predicates were cited. The regulatory pathway of each predicate was also recorded to characterize the lineage structure of contemporary ML-enabled medical devices.

### 2.4. Approval Timeline Evaluation

We calculated the time from FDA submission to clearance for each device by comparing submission and approval dates documented in FDA summaries. These approval timelines were analyzed across different regulatory pathways, medical specialties, and manufacturer origins.

### 2.5. Regulatory Preparedness Evaluation

We assessed the presence of PCCPs in device submissions as an indicator of regulatory preparedness for algorithm modifications. Additionally, we documented mentions of cybersecurity considerations, pediatric indications, and SaMD classifications.

### 2.6. Statistical Analysis

Descriptive statistics are reported as counts and percentages, medians with interquartile ranges (IQR), or means ± SD as appropriate. Continuous variables were compared with the Wilcoxon rank-sum test. Approval-time distributions were right-skewed and therefore summarized with medians (IQR) and compared across pathways and specialty panels with nonparametric tests. Statistical analyses were performed using RStudio (Version 2023.06.1 + 524). A *p*-value < 0.05 was considered statistically significant. Given the primarily descriptive aims of the study, *p*-values are interpreted as heuristic measures of association.

## 3. Results

### 3.1. Characteristics of FDA-Approved AI/ML Medical Devices in 2024

During the calendar year 2024, the FDA approved a total of 168 ML-enabled medical devices. Of these, the vast majority (159 devices, 94.6%) were cleared through the 510(k) regulatory pathway, while 9 devices (5.4%) received approval via the De Novo pathway. All ML-enabled devices authorized in 2024 were classified as Class II under the FDA’s risk-based regulatory framework. Among 510(k) approvals, most were submitted through the Traditional 510(k) process, accounting for 137 devices or 86.2%. An additional 20 devices (12.6%) were cleared via Special 510(k) process and 2 devices (1.3%) via Abbreviated 510(k) (Table 1) process.

### 3.2. Distribution of Devices by Clinical Specialty

**Figure 1:**
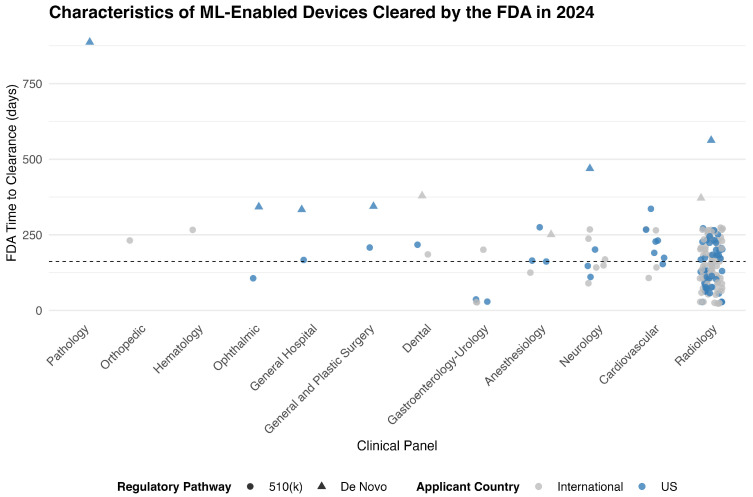
FDA Time to Clearance for ML-Enabled Devices in 2024, by Clinical Panel and Regulatory Pathway. Each point represents a medical device authorized by the FDA in 2024. Symbols indicate regulatory pathway (circle for 510(k), triangle for De Novo), and color indicates applicant origin (blue for U.S.-based sponsors, gray for international). The vertical axis shows the FDA review duration in days. The dashed horizontal line indicates the overall median time to clearance (162 days).

Radiology accounted for the vast majority of ML-enabled device approvals in 2024, with 125 devices (74.4%) cleared under this panel. The next most common specialties were Cardiovascular (11 devices, 6.5%) and Neurology (10 devices, 6.0%). Other panels contributed smaller numbers, including Anesthesiology and Gastroenterology–Urology (5 devices each), Dental (3 devices), and several others with only one or two approvals (Figure 1, Table 1).

### 3.3. Geographic Distribution of FDA-Approved AI/ML Devices

**Figure 2:**
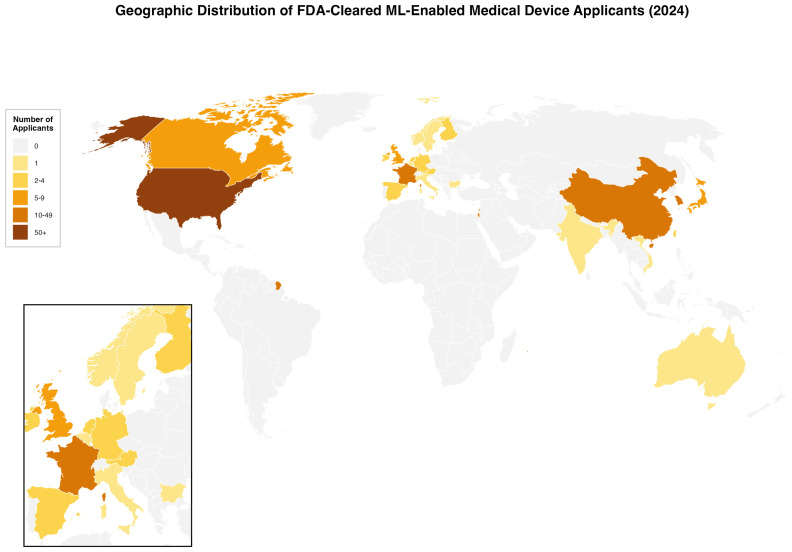
Geographic Distribution of FDA-Cleared ML-Enabled Medical Device Applicants in 2024. World map showing the number of applicants by country for FDA-cleared machine learning-enabled medical devices in 2024 (N = 168). Countries are color-coded by the number of applicants. Inset shows European detail. The US had 71 applicants (42·3%), France 16 (9·5%), and China 14 (8·3%).

In 2024, non-US applicants accounted for the majority of FDA-cleared ML-enabled medical devices, with 97 approvals (57.7%). Overall, 168 devices were cleared from 24 countries. The top contributing countries were the United States (71 devices, 42.3%), followed by France (16, 9.5%), China (14, 8.3%), South Korea (11, 6.5%), Israel (11, 6.5%), and Japan (7, 4.2%) (Figure 2).

### 3.4. Regulatory Genealogy and Innovation Lineage of 510(K) AI/ML Devices

#### 3.4.1. Temporal Patterns of Predicate Selection

**Figure 3:**
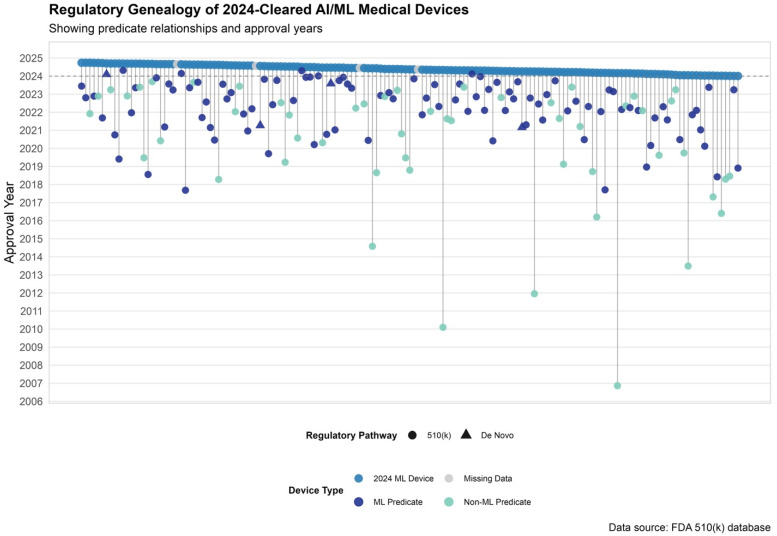
Predicate Genealogy of AI/ML Devices Cleared by the FDA in 2024. Predicate device lineage for 2024-cleared AI/ML-enabled devices. Each vertical line connects a 2024-cleared device (top row) to its primary predicate, color-coded by predicate type: dark blue for ML-enabled predicates, teal for non-ML predicates, and gray for missing data. Shapes indicate regulatory pathway.

Among the 159 AI/ML-enabled devices approved via the 510(k) pathway in 2024, 155 devices (97.5%) had identifiable primary predicates. The remaining 4 devices (2.5%) lacked predicate information in publicly available FDA summaries. Of the 155 devices with valid predicate data, predicates were most commonly approved in 2022 (43 predicates, 27.7%) and 2023 (41 predicates, 26.5%), followed by 2021 (22 predicates, 14.2%) and 2020 (15 predicates, 9.7%). A small number of predicates dated back more than a decade, with the earliest approved in 2006 (Figure 3).

Across all predicate devices cited by AI/ML devices cleared in 2024, the median time since predicate market entry was 2.2 years (IQR: 1.2–4.1). ML-based predicates were significantly newer to market than non-ML predicates (median 1.9 years [IQR: 1.0–3.0] vs. 2.8 years [IQR: 1.7–5.5]; *p* < 0.001).

#### 3.4.2. Predicate Reuse and ML vs. Non-ML Lineage

A total of 141 unique primary predicates were used by 510(k)-cleared AI/ML devices in 2024. Of these, 14 predicates (9.9%) were reused by more than one 2024 device. Among the 141 unique predicates, 91 (64.5%) were identified as AI/ML-based. Eleven of the 91 AI/ML-based predicates (12.1%) were reused, compared to 3 of 50 non-ML predicates (6.0%).

Among reused predicates, the median time from original clearance to reuse was 1.4 years (IQR: 1.2–2.3). ML-based predicates had a median reuse age of 1.5 years (IQR: 1.3–2.2), compared to 1.2 years (IQR: 0.9–4.0) for non–ML predicates.

The majority of predicates were originally cleared through the 510(k) pathway (137 predicates, 97.2%), while 4 predicates (2.8%) were approved via the De Novo pathway (Figure 3).

#### 3.4.3. AI Predicate Lineage Structure

Among the 155 FDA-cleared AI/ML devices approved via the 510(k) pathway in 2024, 126 devices (81.3%) relied solely on a single primary predicate for substantial equivalence. The remaining devices demonstrated more complex regulatory ancestry, with twenty-nine devices (18.7%) citing additional predicates. In addition, 50 devices (32.3%) included reference devices to support technological comparisons.

#### 3.4.4. FDA Approval Timelines

The median time from FDA submission to clearance for all ML-enabled devices in 2024 was 162 days (IQR: 106 to 230 days), with a range of 23 to 887 days. Devices cleared through the 510(k) pathway had a significantly shorter median approval time of 151 days, compared to 372 days for those approved via the De Novo pathway (*p* < 0.001). Across medical specialties, approval times varied substantially. Radiology devices—the most common panel—had a median approval time of 146 days, significantly shorter than devices reviewed under other panels (*p* = 0.002). No statistically significant difference in approval times was observed between devices originating from the United States (median 172 days) and those from international manufacturers (median 151 days) (*p* = 0.42) (Supplementary Table S1).

### 3.5. Performance Metrics and Demographic Representativeness (AI Fairness)

#### 3.5.1. Performance Metrics

Among the 168 ML-enabled medical devices cleared by the FDA in 2024, both sensitivity and specificity were reported jointly in 49 devices (29.2%). Sensitivity alone was reported in 54 devices (32.1%), while specificity alone was reported in 49 devices (29.2%) (Table 2).

#### 3.5.2. Fairness and Representativeness

Demographic information related to sex or race and ethnicity was reported in 77 of the 168 device summaries (45.8%). Sex data were reported in 73 devices (43.5%). Race or ethnicity—categorized as White, Black, Asian, or Other—was reported in 26 devices (15.5%) (Table 2).

### 3.6. Predetermined Change Control Plans and Regulatory Preparedness

Among the 168 AI/ML-enabled medical devices cleared or approved in 2024, 28 devices (16.7%) included PCCPs, while 140 devices (83.3%) did not. Cybersecurity considerations were mentioned in 91 devices (54.2%). Pediatric indications were identified in 30 devices (17.9%). A total of 46 devices (27.4%) were categorized as SaMD (Table 1).

PCCPs were most commonly observed among devices reviewed under the Radiology panel (15 devices), followed by Neurology (4 devices), Anesthesiology (3 devices), and smaller counts in other specialties. By region, US-based manufacturers accounted for 17 of 28 PCCPs (60.7%), while 11 devices (39.3%) originated from international developers, including submissions from Israel (3 devices, 10.7%) and France (2 devices, 7.1%). Overall, PCCPs were submitted by manufacturers from nine different countries.

## 4. Discussion

In 2024, the FDA authorized a record 168 AI/ML-enabled devices, surpassing prior years and signaling sustained momentum in digital health innovation [12]. Radiology remained dominant, but nearly 58% of approvals originated from international sponsors. While the majority of devices were cleared via the 510(k) pathway, most predicates were recent and increasingly AI/ML-enabled, reflecting the fast-paced evolution of this sector. Despite regulatory advances, the actual uptake of PCCPs and performance transparency remains modest. Only 16.7% of devices reported PCCPs, and less than one-fifth disclosed complete sensitivity/specificity metrics. Demographic data reporting improved from historical norms but still fell short: just 15.5% of summaries reported race or ethnicity, limiting the ability to evaluate representativeness and equity. To our knowledge, this is the first study to systematically evaluate PCCP reporting in FDA-authorized AI/ML-enabled medical devices following the implementation of the FDA’s 2024 PCCP guidance.

These findings have several regulatory-science implications. The modest uptake of PCCPs, despite their importance for lifecycle oversight, suggests ongoing uncertainty among manufacturers about how to operationalize adaptive model management under emerging FDA expectations [9,13]. Similarly, limited reporting of performance metrics and demographic characteristics aligns with prior evidence of substantial transparency gaps in FDA-cleared AI technologies [1] and reinforces concerns about inadequate information for assessing clinical generalizability at the time of authorization [14]. Persistent underreporting of race and ethnicity is especially consequential given growing evidence of significant subgroup performance differences across diverse populations [15]. Taken together, these patterns show that key disclosure practices remain incomplete and have not yet kept pace with evolving regulatory expectations.

Compared with the 1995–2023 baseline of 692 devices, the 2024 cohort showed a steeper year-to-year growth rate, shorter review times, and slightly improved transparency. Demographic reporting rose from 3.6% to 16%, yet fewer than one in five summaries provided race or ethnicity data [1]. While this reflects progress, the absence of structured, subgroup-level reporting continues to limit assessments of fairness and external validity in healthcare AI. Persistent gaps in demographic transparency reflect entrenched representation bias—a well-documented barrier to the generalizability of model performance and clinical outcomes across diverse populations [16,17].

Recent evaluations further highlight the scale of this challenge. A systematic review of 48 AI studies found that more than half exhibited a high risk of bias, largely attributable to absent sociodemographic data, imbalanced datasets, and inadequate algorithmic design [18]. Similarly, a review of 555 neuroimaging AI models revealed that 83% had a high risk of bias, with most studies relying almost exclusively on data from high-income regions [19]. Likewise, a systematic review demonstrated that among 11 cardiovascular AI studies reviewed, 9 (82%) showed significant performance differences across racial and ethnic groups, with some studies showing sensitivity differences as high as 52.6% versus 39.6% between Black and White patients [15].

Our findings reinforce prior evidence that representation bias is not an isolated anomaly but a pervasive, systemic flaw. Combined with algorithmic biases introduced during model development and validation, representation bias critically undermines the generalizability and clinical applicability of AI/ML models. Without systematic strategies for bias recognition and mitigation, the ethical and equitable deployment of AI in healthcare will remain at risk.

Consistent with the historical dominance of the substantial-equivalence pathway [20,21], the overwhelming majority of AI/ML-enabled medical devices cleared in 2024 proceeded through the 510(k) pathway. Despite the innovative nature of AI/ML devices and their moderate-risk classification, De Novo review remained limited to five percent of all approvals. The markedly shorter review times and lower evidentiary thresholds associated with 510(k) submissions likely further incentivized manufacturers to pursue the 510(k) route.

Analysis of primary predicate genealogy revealed that more than one-third of devices cleared via 510(k) relied on conventional, non-ML-based systems, similar to trends observed between 2019 and 2021 [22]. Predicate reuse was uncommon—only 14 of 141 unique predicates (9.9%) were cited by more than one 2024 clearance—indicating a rapidly turning lineage in which most predicates serve a single subsequent device. These findings highlight a persistent reliance on the 510(k) framework, even for software that is ostensibly innovative, and reveal a mixed predicate ancestry that may complicate future efforts to monitor safety signals across related AI products.

Beyond the substantial-equivalence framework, other regulatory domains such as cybersecurity and change management also merit attention. Although cybersecurity considerations were mentioned in just over half of device summaries and PCCPs appeared in only 17% of cases, both findings likely reflect the recency of regulatory expectations in these areas. As PCCP frameworks and cybersecurity guidance continue to evolve, improvements in the completeness and consistency of these disclosures may be anticipated in future device cohorts. As the global development pipeline for AI/ML-enabled medical devices continues to expand [23], proactive and adaptive regulatory strategies will be essential to ensure that future approvals adequately address emerging clinical and technological complexity.

The study has some limitations. First, the analysis relied exclusively on publicly available FDA summaries, which often lack standardized reporting, particularly regarding performance metrics, demographic representativeness, and cybersecurity measures. This limitation may have led to an underestimation of the true extent of disclosures, such as PCCPs or risk mitigation strategies. Second, the classification of devices as AI/ML-based depended on available documentation; in cases of ambiguous or incomplete descriptions, there is a possibility of misclassification. Third, the study could not assess postmarket modifications, iterative algorithm updates, or real-world performance drift, which are increasingly relevant in evaluating the lifecycle safety and effectiveness of AI/ML-enabled medical devices. Fourth, while the analysis captures regulatory genealogy through primary predicate selection, it does not trace extended secondary or tertiary generation predicate linkages, potentially underestimating the complexity of predicate ancestry. Finally, given the recent introduction of PCCP and cybersecurity frameworks, findings from the 2024 cohort may not fully reflect the long-term uptake or impact of these regulatory initiatives, and ongoing surveillance will be necessary to assess trends over time.

## 5. Conclusions

In 2024, the FDA approved a record number of ML-enabled medical devices, with the vast majority cleared via the 510(k) pathway. Approvals were predominantly concentrated in radiology and increasingly originated from international developers. While most devices relied on recently approved predicates, predicate reuse remained uncommon. Interestingly, reused predicates were more frequently AI/ML-based than non-AI/ML-based. Approval timelines were shortest for specialties with the highest volume of submissions. Reporting of performance metrics and demographic representation was inconsistent. Additionally, cybersecurity considerations and Predetermined Change Control Plans were documented in only a minority of devices. A substantial proportion of submissions were classified as software as a medical device, underscoring the increasing digitalization of regulated clinical technologies.

Future work should track how disclosure practices evolve over time and whether emerging FDA initiatives, such as PCCPs and strengthened cybersecurity expectations, improve the completeness and quality of public information. Longitudinal studies linking regulatory data with clinical and postmarket performance will be essential to determine whether current pathways adequately support the safe, effective, and equitable deployment of medical AI.

## Figures and Tables

**Table 1:** Summary Characteristics of Machine Learning-Enabled Medical Devices Approved by the FDA in 2024 (n = 168).

Characteristic	No. (%)
**Regulatory Pathway**	
510(k) premarket notification	159 (94.6%)
De Novo classification	9 (5.4%)
**FDA Review Panel**	
Radiology Devices	125 (74.4%)
Cardiovascular Devices	11 (6.5%)
Neurology Devices	10 (6.0%)
Anesthesiology Devices	5 (3.0%)
Gastroenterology–Urology	5 (3.0%)
Dental	3 (1.8%)
General and Plastic Surgery	2 (1.2%)
General Hospital	2 (1.2%)
Ophthalmic	2 (1.2%)
Other\††	3 (1.8%)
**Review Time, days\‡**	
Median (IQR)	162 (106–230)
Range	23 to 887
**Developer Country**	
Outside the United States	97 (57.7%)
United States	71 (42.3%)
**Pediatric Indication**	
Indicated for pediatric use	30 (17.9%)
Not indicated	138 (82.1%)
**Cybersecurity Mentioned**	
Yes	91 (54.2%)
No	77 (45.8%)
**PCCP Mentioned**	
Yes	28 (16.7%)
No	140 (83.3%)
**SaMD**	
Yes	46 (27.4%)
No	122 (72.6%)

\‡ Calculated using dates of submission and decision as listed in the FDA database. \†† Hematology, Orthopedic, Pathology each 1 (0.6%).

**Table 2:** Regulatory and Clinical Characteristics of FDA-Authorized Machine Learning-Enabled Medical Devices in 2024.

Device Name	FDA Number	Regulatory Pathway	Reported Algorithm	Intended Use	Performance Metrics		Demographic Representation				
					Sensitivity (%)	Specificity (%)	Female (%)	White (%)	Black (%)	Asian (%)	Other (%)
**Anesthesiology**											
Aurora	K231355	510(k)	Deep Learning	Medical image visualization and annotation	89.4 to 92.6 *	71.6 to 76.8 *	NR	NR	NR	NR	NR
Huxley SANSA Home Sleep Apnea Test (1000-00)	K240285	510(k)	AI	Obstructive sleep apnea diagnosis	88.2	87.3	NR	NR	NR	NR	NR
Sleep Apnea Feature	DEN230041	De Novo	Machine Learning	Sleep apnea detection	82.7	87.7	48.1	NR	NR	NR	NR
Sleep Apnea Notification Feature (SANF)	K240929	510(k)	Deep Learning	Sleep apnea risk assessment	66.3	98.5	56.5	68.1	23.1	6.9	1.88
Tyto Insights for Crackles Detection	K240555	510(k)	Neural Network	Crackles detection for respiratory disease	72	99	52.2	NR	NR	NR	NR
**Cardiovascular**											
APPRAISE-HRI	K233249	510(k)	Machine Learning	Liver disease risk prediction	NR	NR	NR	NR	NR	NR	NR
ASSURE Wearable ECG	K233864	510(k)	Other	Atrial fibrillation detection via wearable ECG	NR	NR	NR	NR	NR	NR	NR
Acumen Assisted Fluid Management (AFM) Software Feature	K233984	510(k)	Other	Fluid management optimization in critical care	NR	NR	NR	NR	NR	NR	NR
CLEWICU System	K233216	510(k)	Machine Learning	ICU patient deterioration prediction	63 to 69 *	87 to 93 *	49	NR	NR	NR	NR
CorVista System with PH Add-On	K233666	510(k)	Machine Learning	Pulmonary hypertension assessment	82	92	NR	NR	NR	NR	NR
EchoGo Heart Failure (2.0)	K240013	510(k)	CNN	Heart failure risk assessment	90.3	86.1	NR	NR	NR	NR	NR
Eko Low Ejection Fraction Tool (ELEFT)	K233409	510(k)	Deep Learning	Low ejection fraction detection from heart sounds	74.7	77.5	44.3	58.2	21.6	15	4.3
HeartKey^®^ Rhythm	K233755	510(k)	Machine Learning	Cardiac arrhythmia detection	NR	NR	16	NA	NR	NR	24 non-White; 60 Unknown
Impala	K231010	510(k)	Deep Learning	Cardiac monitoring and data transmission	NR	NR	48	56	33	2.2	0.1
Tempus ECG-AF	K233549	510(k)	Machine Learning	Atrial fibrillation detection from ECG	31	92	56	82	11	3	4 Other; 1 Unknown
eCARTv5 Clinical Deterioration Suite (“eCART”)	K233253	510(k)	Machine Learning	Hospital deterioration prediction and early warning	38.6 to 51.8 *	93.1 to 96.9 *	NR	79.2	12.6	1.9	0.4 AI/AN; 0.2 NH/PI; 5.8 Unknown
**Dental**											
DentalMonitoring	DEN230035	De Novo	Neural Network	Remote orthodontic monitoring software	79.9 to 100 *	83.2 to 100 *	28.8 to 68.6 *	70 to 88.4 *	4.8 to 18 *	2.1 to 4.7 *	3 to 6 AI/AN *
RAYDENT SW	K233625	510(k)	Machine Learning	Dental 3D modeling and prosthesis design	NR	NR	NR	NR	NR	NR	NR
X-Guide Surgical Navigation System	K232148	510(k)	Machine Learning	Dental surgical navigation	NR	NR	NR	NR	NR	NR	NR
**Gastroenterology**–**Urology**											
CADDIE	K240044	510(k)	AI	Colorectal polyp detection	98.3	98.5	45	85.2	3.2	6.76	0.1 AI/AN; 1.9 NH/PI; 3.0 Other
GI Genius™ Module 100 (GGM100.US); GI Genius™ Module 200 (GGM200.US); ColonPRO™ 4.0 (CPRO40.US)	K233964	510(k)	Neural Network	Colorectal polyp detection during colonoscopy	88.1	NR	NR	NR	NR	NR	NR
Genius™ Module 100 (GGM100.US); GI Genius™ Module 200 (GGM200.US); ColonPRO™ 4.0 (CPRO40.US); GI Genius™ Module 300 (GGM300-US); ColonPRO™ 4.0 (CPRO40S-US)	K241887 †	510(k)	Missing File	Missing File	Missing File	Missing File	Missing File	Missing File	Missing File	Missing File	Missing File
SKOUT^®^ system	K241508	510(k)	AI	Colorectal polyp detection	NR	NR	NR	NR	NR	NR	NR
SKOUT^®^ system	K240781	510(k)	AI	Colorectal polyp detection	NR	NR	NR	NR	NR	NR	NR
**General Hospital**											
Sepsis ImmunoScore	DEN230036	De Novo	Machine Learning	Sepsis risk prediction	36 to 67 *	90 to 94 *	43.7	57.6	22.7	2.1	0.3 AI/AN; 0.1 NH/PI; 17.2 Unknown
SurgiCount+ System	K232250	510(k)	AI	Surgical item counting and tracking	NR	NR	NR	NR	NR	NR	NR
**General and Plastic Surgery**											
DermaSensor	DEN230008	De Novo	Neural Network	Skin cancer risk assessment	95.5	20.7	51.5	97.1	7	8.9	1.0 Multiracial/NH/PI
HYDROS Robotic System; HYDROS TRUS Probe; HYDROS Handpiece	K240200	510(k)	AI	Prostate cancer biopsy guidance	NR	NR	NR	NR	NR	NR	NR
**Hematology**											
AUTION EYE AI-4510 Urine Particle Analysis System	K232416	510(k)	Other	Urinary tract condition detection via urine analysis	76.2	83.7	NR	NR	NR	NR	NR
**Neurology**											
ARVIS^®^ Shoulder	K240062	510(k)	Deep Learning	Shoulder replacement surgery navigation	NR	NR	55	NR	NR	NR	NR
Automatic Registration	K234047	510(k)	CNN	Image registration for radiological analysis	NR	NR	NR	NR	NR	NR	NR
BrainSee	DEN220066	De Novo	AI	Alzheimer’s disease detection and monitoring	72.9	96.3	44.9	89.9	3.5	3.5	2.5 Multiracial
EpiMonitor	K232915	510(k)	Other	Seizure detection and monitoring	89.5 to 98.8 *	NR	53.1	NR	NR	NR	NR
OTS Hip	K232140	510(k)	Machine Learning	Hip surgery planning	NR	NR	NR	NR	NR	NR	NR
Oxevision Sleep Device	K233618	510(k)	NR	Sleep monitoring and assessment	>88% **	>55 **	50.0	NR	NR	NR	NR
REMI AI Discrete Detection Module	K231779	510(k)	Machine Learning	COVID-19 detection from chest X-rays	86.2	NR	48.0	NR	NR	NR	NR
SleepStageML	K233438	510(k)	Deep Learning	Sleep stage classification for sleep disorders	NR	NR	46.0	NR	NR	NR	NR
autoSCORE	K231068	510(k)	Neural Network	Heart failure management using EHR data	83.1 to 100 *	91.8 to 94.4 *	47.2	NR	NR	NR	NR
encevis (2.1)	K240993	510(k)	NR	Seizure monitoring and analysis	71.6	NR	NR	NR	NR	NR	NR
**Ophthalmic**											
AEYE-DS	K240058	510(k)	AI	Diabetic retinopathy detection	92 to 93 *	89 to 94 *	49 to 55 *	46 to 47 *	19 to 26 *	NR	22 to 30 Hispanic or Latino *: remainder other
Notal Vision Home Optical Coherence Tomography (OCT) System	DEN230043	De Novo	AI	Home monitoring of retinal diseases via OCT	86.4	84.9	56.3 to 58.6 *	95.8 to 96.9 *	1.5 to 1.9 *	0.3 to 0.6 *	0.3 AI/AN; 0.3 NH/PI; 1.8 Unknown
**Orthopedic**											
Precision AI Surgical Planning System (PAI-SPS)	K233992	510(k)	Deep Learning	Surgical planning for orthopedic conditions	NR	NR	NR	NR	NR	NR	NR
**Pathology**											
“Genius™ Digital Diagnostics System With The Genius™ Cervical AI Algorithm”	DEN210035	De Novo	CNN	Cervical cancer screening using AI-assisted cytology	66.7 to 91.7 *	91 to 98.5 *	52	NR	NR	NR	NR
**Radiology**											
ACUSON Sequoia Diagnostic Ultrasound System, ACUSON Sequoia Select Diagnostic Ultrasound System, ACUSON Origin Diagnostic Ultrasound System, ACUSON Origin ICE Diagnostic Ultrasound System	K240704	510(k)	NR	General ultrasound diagnostic imaging	NR	NR	23	NR	NR	NR	NR
ADAS 3D	K240791	510(k)	Machine Learning	Cardiac 3D imaging analysis	NR	NR	NR	NR	NR	NR	NR
AI Platform 2.0 (AIP002)	K240953	510(k)	CNN	Prostate cancer risk prediction	NR	NR	NR	NR	NR	NR	NR
AI-Rad Companion (Pulmonary)	K233753	510(k)	Deep Learning	Pulmonary nodule analysis and triage	NR	NR	53	NR	NR	NR	NR
AI-Rad Companion Organs RT	K232899	510(k)	Deep Learning	Organ contouring for radiotherapy planning	NR	NR	16.61 to 52.5 *	NR	NR	NR	NR
AISAP Cardio V1.0	K234141	510(k)	Neural Network	Cardiac function assessment	86.5 to 96.5 *	89.3 to 97 *	36.8	77.5	13	5.5	4 Hispanic or Latino
ART-Plan (v.2.2.0)	K234068	510(k)	Deep Learning	Radiotherapy planning for cancer treatment	NR	NR	NR	NR	NR	NR	NR
AVIEW CAC	K233211	510(k)	Deep Learning	Coronary artery calcium scoring	NR	NR	NR	NR	NR	NR	NR
Acorn 3D Software (AC-SEG-4009); Acorn 3DP Model (AC-101-XX)	K234009	510(k)	Machine Learning	3D modeling for orthopedic surgical planning	NR	NR	NR	NA	NR	NR	NR
AiMIFY (1.x)	K240290	510(k)	Neural Network	Lung nodule malignancy risk prediction	NR	NR	NR	NR	NR	NR	NR
Aplio i900, Aplio i800 and Aplio i700 Software V8.1 Diagnostic Ultrasound System	K233195	510(k)	NR	Ultrasound image visualization and analysis	NR	NR	52.2	NR	NR	NR	NR
Aplio i900/i800/i700 Diagnostic Ultrasound System, Software V7.0 (TUS-AI900, TUS-AI800, TUS-AI700)	K241582	510(k)	Deep Learning	General ultrasound diagnostic system	NR	NR	50	NR	NR	NR	NR
Aquilion ONE (TSX-308A/3) V1.4 with PIQE Reconstruction System	K232835	510(k)	CNN	CT imaging with enhanced reconstruction	NR	NR	NR	NR	NR	NR	NR
Biograph VK10	K233677	510(k)	NR	PET/CT imaging for oncology and cardiology	NR	NR	NR	NR	NR	NR	NR
BoneMRI	K233030	510(k)	CNN	Bone and musculoskeletal MRI analysis	NR	NR	25 to 51	NR	NR	NR	NR
BriefCase-Quantification	K241112	510(k)	Deep Learning	Lung lesion quantification and analysis	NR	NR	53.1	NR	NR	NR	NR
BriefCase-Triage	K241727	510(k)	Deep Learning	Pulmonary embolism triage and notification	NR	NR	NR	NR	NR	NR	NR
Butterfly iQ3 Ultrasound System	K232808	510(k)	CNN	General ultrasound diagnostic imaging	NR	NR	NR	NR	NR	NR	NR
CAC (gated) Algorithm	K240369	510(k)	NR	Coronary artery calcium assessment	NR	NR	NR	NR	NR	NR	NR
CEPHX- Cephalometric Analysis Software	K231396	510(k)	NR	Cephalometric analysis for orthodontics	NR	NR	NR	NR	NR	NR	NR
CINA-ASPECTS	K233342	510(k)	Deep Learning	Stroke severity assessment using ASPECTS score	NR	NR	NR	NR	NR	NR	NR
CINA-CSpine	K240942	510(k)	Deep Learning	Cervical spine fracture detection	90.3	91.9	36.3	NR	NR	NR	NR
CINA-VCF	K240612	510(k)	Deep Learning	Vertebral compression fracture triage and detection	95.2	92.9	50.8	NR	NR	NR	NR
CINA-iPE	K233968	510(k)	Deep Learning	Pulmonary embolism triage and detection	87.8	92	46.7	NR	NR	NR	NR
CT 5300	K232491	510(k)	Deep Learning	General CT diagnostic imaging	NR	NR	NR	NR	NR	NR	NR
CT Cardiomegaly	K232613	510(k)	Machine Learning	Cardiomegaly detection from CT	NR	NR	48.4	NR	NR	NR	NR
CardIQ Suite	K233731	510(k)	Deep Learning	Cardiac imaging and analysis	NR	NR	NR	NR	NR	NR	NR
Cardiac CT Function Software Application	K241038	510(k)	Machine Learning	Cardiac function analysis from CT	NR	NR	NR	NR	NR	NR	NR
Clarius OB AI	K233955	510(k)	Deep Learning	Obstetric ultrasound fetal measurement	NR	NR	100	NR	NR	NR	NR
CoLumbo	K241211	510(k)	Deep Learning	Colon polyp assessment	NR	NR	NR	NR	NR	NR	NR
Constellation (CON-001)	K241280	510(k)	CNN	Pulmonary embolism triage and notification	NR	NR	NR	NR	NR	NR	NR
DASI Dimensions (V1.0)	K231324	510(k)	Deep Learning	Cardiac risk stratification from EHR data	85.3	NR	45	NR	NR	NR	NR
DeepContour (V1.0)	K232928	510(k)	Deep Learning	Head and neck tumor segmentation	NR	NR	53.5 to 57 *	NR	NR	NR	NR
ECHELON Synergy V10.0	K233687	510(k)	Deep Learning	MRI imaging system for whole-body diagnostics	NR	NR	36.4	NR	NR	NR	NR
EFAI Bonesuite XR Bone Age Pro Assessment System (BAP-XR-100)	K234042	510(k)	Deep Learning	Pediatric bone age assessment	NR	NR	50	NR	NR	NR	NR
EFAI CARDIOSUITE CTA ACUTE AORTIC SYNDROME ASSESSMENT SYSTEM	K240291	510(k)	AI	Aortic syndrome detection from CTA	92.9	91.5	51.6	NR	NR	NR	NR
EPIQ Series Diagnostic Ultrasound System, Affiniti Series Diagnostic Ultrasound System	K233788	510(k)	AI	General ultrasound diagnostic imaging	NR	NR	56.3	31.3	53.8	2.8	9.5
EPIQ Series Diagnostic Ultrasound Systems; Affiniti Series Diagnostic Ultrasound Systems	K240850	510(k)	Deep Learning	General ultrasound diagnostic imaging	NR	NR	NR	NR	NR	NR	NR
EdgeFlow UH10	K231677	510(k)	Neural Network	Blood flow visualization in ultrasound imaging	NR	NR	62.2	NR	NR	NR	NR
Ethos Treatment Management (3.0); Ethos Treatment Planning (2.0)	K232923	510(k)	Neural Network	Oncology treatment planning and management	NR	NR	NR	NR	NR	NR	NR
FETOLY-HEART	K241380	510(k)	Deep Learning	Fetal cardiac function analysis	98.3 to 100 *	99.8	100	56.6	26.5	4.7	12.2
Fibresolve	DEN220040	De Novo	Deep Learning	Liver fibrosis quantification	41	87	46.7	81.8	10.9	2.1	5.1
HealthCCSng	K241440	510(k)	Deep Learning	Coronary artery calcium scoring	NR	NR	41.7	NR	NR	NR	NR
HealthFLD	K233080	510(k)	Deep Learning	Fatty liver disease detection from imaging	NR	NR	49	NR	NR	NR	NR
Heuron ICH	K233247 †	510(k)	Missing File	Missing File	Missing File	Missing File	Missing File	Missing File	Missing File	Missing File	Missing File
Hyper Insight—ICH	K240353	510(k)	Deep Learning	Intracranial hemorrhage triage and notification	95.5	98.5	NR	NR	NR	NR	NR
Imbio PHA (4.0.0)	K241847	510(k)	Deep Learning	Pulmonary hypertension assessment	NR	NR	NR	NR	NR	NR	NR
InVision Precision LVEF (LVEF)	K232331	510(k)	NR	Left ventricular ejection fraction estimation	NR	NR	NR	NR	NR	NR	NR
JBS-LVO	K241480	510(k)	CNN	Large vessel occlusion triage and notification	91.8	92.8	NR	NR	NR	NR	NR
Kosmos	K233826	510(k)	NR	Cardiac, lung, and abdominal ultrasound analysis	NR	NR	NR	NR	NR	NR	NR
LVivo IQS	K240769	510(k)	AI	Left ventricular function analysis	NR	NR	NR	NR	NR	NR	NR
LumiNE US; Lumi	K240094	510(k)	Machine Learning	Lung nodule visualization and assessment	NR	NR	NR	NR	NR	NR	NR
LungQ v3.0.0	K232412	510(k)	NR	Pulmonary function assessment in lung disease	NR	NR	NR	NR	NR	NR	NR
MAGNETOM Cima.X Fit	K232765	510(k)	Deep Learning	High-resolution MRI diagnostic imaging	NR	NR	NR	NR	NR	NR	NR
MAGNETOM Terra; MAGNETOM Terra.X	K232322	510(k)	Deep Learning	High-field MRI system for head/extremity imaging and spectroscopy to assist diagnosis.	NR	NR	56	NR	NR	NR	NR
MI View&GO	K242300	510(k)	NR	Molecular imaging visualization	NR	NR	NR	NR	NR	NR	NR
MICSI-RMT	K241121	510(k)	Machine Learning	Brain imaging in multiple sclerosis	NR	NR	NR	NR	NR	NR	NR
MammoScreen^®^ (3)	K240301	510(k)	Deep Learning	Breast cancer detection from mammograms	79.3	83.6	NR	37	16	18	0.8 AI/AN; 0.2 NH/PI; 28 Unknown
Medihub Prostate	K233196	510(k)	Deep Learning	Prostate cancer imaging and contouring	NR	NR	0	92.9	6.1	0.9	NR
NAEOTOM Alpha	K233657	510(k)	NR	General CT diagnostic imaging	NR	NR	NR	NR	NR	NR	NR
NemoScan	K232698	510(k)	NR	Dental implant planning	NR	NR	NR	NR	NR	NR	NR
NeuroQuant	K241098	510(k)	Deep Learning	Brain volume quantification for neurological conditions	NR	NR	42 to 47	87.8	2.9	1.5	1.8 Multiracial; 5.7 Unknown
O-arm O2 Imaging System	K240465	510(k)	Deep Learning	Intraoperative spinal imaging	NR	NR	NR	92.9	6.1	8.9	NR
OptimMRI (v2)	K242054	510(k)	Machine Learning	MRI image optimization and quality enhancement	NR	NR	NR	NR	NR	NR	NR
Overjet Caries Assist-Pediatric	K233738	510(k)	Machine Learning	Dental caries detection in pediatric patients	83.9	97.5	NR	NR	NR	NR	NR
Overjet Charting Assist	K241684	510(k)	NR	Dental radiograph analysis and charting	NR	NR	NR	NR	NR	NR	NR
Overjet Charting Assist	K233590	510(k)	AI	Dental radiograph charting and annotation	79.9 to 95.9 *	86.3 to 99.9 *	53.15	NR	NR	NR	NR
Overjet Image Enhancement Assist	K241681	510(k)	Machine Learning	Dental condition imaging enhancement	NR	NR	NR	NR	NR	NR	NR
PIUR tUS Infinity	K240036	510(k)	Machine Learning	Thyroid disease diagnosis	NR	NR	NR	NR	NR	NR	NR
Preview Shoulder	K240172	510(k)	NR	Shoulder surgery planning	NR	NR	NR	NR	NR	NR	NR
QOCA^®^ image Smart RT Contouring System	K231855	510(k)	Deep Learning	Radiotherapy organ contouring	NR	NR	44.1	NR	NR	NR	NR
RS85 Diagnostic Ultrasound System	K240516	510(k)	Deep Learning	Musculoskeletal ultrasound imaging	NR	NR	50 to100 *	NR	NR	NR	NR
RUS	K233457	510(k)	Machine Learning	Orthopedic imaging and templating	NR	NR	NR	NR	NR	NR	NR
Radify Triage	K231871	510(k)	CNN	Chest X-ray triage for lung conditions	94.4 to 94.8 *	96.4 to 97.9 *	45.4	NR	NR	NR	NR
Radiography 7300 C	K233662 †	510(k)	Missing File	Missing File	Missing File	Missing File	Missing File	Missing File	Missing File	Missing File	Missing File
Rapid	K233582	510(k)	AI	Stroke imaging and perfusion assessment	NR	NR	NR	NR	NR	NR	NR
Rapid (6.0)	K233512	510(k)	Machine Learning	Stroke imaging and perfusion analysis	NR	NR	NR	NR	NR	NR	NR
Rapid ASPECTS (v3)	K232156	510(k)	Machine Learning	Stroke severity assessment using ASPECTS score	NR	NR	52	NR	NR	NR	NR
Rayvolve	K240845	510(k)	CNN	Fracture detection in extremities	95.5	83.1	NR	NR	NR	NR	NR
Relu Creator	K233925	510(k)	Other	Radiograph enhancement for diagnosis	NR	NR	NR	NR	NR	NR	NR
Revolution Ascend Sliding	K233749	510(k)	NR	General CT diagnostic imaging	NR	NR	NR	NR	NR	NR	NR
Rho	DEN230023	De Novo	Machine Learning	Radiology report generation	36 to 67 *	90 to 94 *	NR	NR	NR	NR	NR
SIGNA Champion	K233728	510(k)	NR	General MRI diagnostic imaging	NR	NR	NR	NR	NR	NR	NR
SIS System	K241083	510(k)	Machine Learning	Orthopedic surgical planning	NR	NR	NR	NR	NR	NR	NR
SMART Bun-Yo-Matic CT	K240642	510(k)	Machine Learning	Chest CT image reconstruction	100	98	NR	NR	NR	NR	NR
SMART Bun-Yo-Matic X-Ray	K240736	510(k)	NR	Chest X-ray analysis for lung conditions	NR	NR	NR	NR	NR	NR	NR
SOMATOM go.Up; SOMATOM go.Now; SOMATOM go.All; SOMATOM go.Top; SOMATOM go.Sim; SOMATOM go.Open Pro; SOMATOM X.cite; SOMATOM X.ceed	K233650	510(k)	CNN	General CT diagnostic imaging	NR	NR	NR	NR	NR	NR	NR
See-Mode Augmented Reporting Tool, Thyroid (SMART-T)	K240697	510(k)	Machine Learning	Thyroid ultrasound characterization	NR	NR	81.5	NR	NR	NR	NR
SmartChest	K232410	510(k)	CNN	Chest X-ray triage and pulmonary condition detection	92.7 to 93.3 *	90 to 97.3 *	44.3 to 47.3 *	NR	NR	NR	NR
Sonio Detect	K240406	510(k)	AI	Fetal ultrasound anomaly detection	86.1 to 98.7 *	85.6 to 98.7 *	NR	NR	NR	NR	NR
SubtleSYNTH (1.x)	K241329	510(k)	CNN	MRI image enhancement for brain imaging	NR	NR	NR	NR	NR	NR	NR
Swoop^®^ Portable MR Imaging^®^ System	K240944	510(k)	Deep Learning	Brain MRI imaging at point of care	NR	NR	NR	NR	NR	NR	NR
Syngo Carbon Enterprise Access (VA40A)	K240294	510(k)	NR	Enterprise imaging access for diagnostics	NR	NR	NR	NR	NR	NR	NR
TRAQinform IQ	K233998	510(k)	Machine Learning	Cancer therapy response assessment	78	36	55.3	24.3	1	NR	1.9 Hispanic; 72.8 Unknown
True Enhance DL	K233698	510(k)	Deep Learning	MRI image enhancement with deep learning	NR	NR	NR	NR	NR	NR	NR
Us2.v2	K233676	510(k)	NR	Ultrasound image processing for cardiovascular analysis	NR	NR	45.8 to 55.8 *	18.8 to 87.9 *	3.7 to 25 *	NR	0 to 25.8 Hispanic *; 7.7 to 13.4 Other *
V8/XV8/XH8, V7/XV7/XH7, V6/XV6/XH6 Diagnostic Ultrasound System	K240631	510(k)	AI	General diagnostic ultrasound imaging	NR	NR	NR	NR	NR	NR	NR
VEA Align	K231917	510(k)	Machine Learning	Orthopedic alignment planning	NR	NR	NR	NR	NR	NR	NR
VEA Align; spineEOS	K240582	510(k)	Machine Learning	Spinal alignment planning and analysis	NR	NR	NR	NR	NR	NR	NR
Vantage Fortian/Orian 1.5T, MRT-1550, V9.0 with AiCE Reconstruction Processing Unit for MR	K240238	510(k)	Deep Learning	General MRI imaging with image enhancement	NR	NR	50 to 73.3	NR	NR	NR	NR
Vantage Galan 3T, MRT-3020, V10.0 with AiCE Reconstruction Processing Unit for MR	K241496	510(k)	Deep Learning	General MRI imaging and reconstruction	NR	NR	NR	NR	NR	NR	NR
Velacur	K233977	510(k)	CNN	Liver stiffness estimation	>80% **	>80% **	75.7	69	NR	NR	31 non-White
Velmeni for Dentists (V4D)	K240003	510(k)	Neural Network	Dental radiographic condition detection	72.8	88	NR	NR	NR	NR	NR
VinDr-Mammo	K233108	510(k)	AI	Breast cancer triage from mammography	90	91	100	NR	NR	NR	NR
VisAble.IO	K240773	510(k)	AI	CT and MR imaging visualization	NR	NR	26.86 to 34.58 *	NR	NR	NR	NR
Viz HDS, Viz Volume Plus, Viz ICH+	K232363	510(k)	Machine Learning	Stroke triage including hemorrhage and volume	96	98.4	49	NR	NR	NR	NR
Voluson Signature 20, Voluson Signature 18	K233692	510(k)	NR	Obstetric ultrasound fetal imaging	NR	NR	NR	NR	NR	NR	NR
YSIO X.pree	K233543	510(k)	AI	General X-ray imaging system	NR	NR	NR	NR	NR	NR	NR
i2Contour	K233822	510(k)	AI	Oncology tumor contouring for radiation planning	92.7 to 93.4 *	97.2 to 98.6 *	NR	NR	NR	NR	NR
iCAS-LV	K231690	510(k)	Deep Learning	Left ventricular function analysis from CT	NR	NR	47.2	NR	NR	NR	NR
inHEART Models	K231683	510(k)	Neural Network	Cardiac anatomy modeling for arrhythmia treatment	NR	NR	NR	NR	NR	NR	NR
qCT LN Quant	K240740	510(k)	Deep Learning	Lung nodule quantification in oncology	NR	NR	NR	NR	NR	NR	NR
syngo.CT Brain Hemorrhage	K232431	510(k)	AI	Intracranial hemorrhage detection from CT	86.1 to 95 *	85.2 to 93.1 *	NR	NR	NR	NR	NR
syngo.via MI Workflows; Scenium; syngo MBF	K242275	510(k)	NR	Molecular imaging workflow support	92	96.3	42.6	NR	NR	NR	NR
syngo.via RT Image Suite	K232799	510(k)	Deep Learning	Radiotherapy imaging planning and analysis	NR	NR	42.1	NR	NR	NR	NR
uAI Easy Triage ICH	K242292	510(k)	CNN	Intracranial hemorrhage triage and notification	92	95	51	63	18	NR	19
uAI Portal	K240411	510(k)	AI	Clinical data integration and workflow support	NR	NR	48.7	NR	NR	NR	NR
uMI Panorama	K241585	510(k)	CNN	General PET/CT diagnostic imaging	NR	NR	46.2	NR	NR	NR	NR
uMI Panvivo	K241596	510(k)	Deep Learning	Whole-body PET/CT imaging for oncology and neurology	NR	NR	NR	NR	NR	NR	NR
uMR 680	K240744	510(k)	NR	General diagnostic MRI imaging	NR	NR	NR	NR	NR	NR	NR
uMR Jupiter	K233673	510(k)	AI	General MRI diagnostic imaging	NR	NR	40	16	NR	84	NR
uMR Omega	K240540	510(k)	NR	Whole-body MRI diagnostic imaging	NR	NR	NR	NR	NR	NR	NR
uOmnispace.CT	K233209	510(k)	Deep Learning	CT imaging visualization and manipulation	NR	NR	35 to 41.66 *	NR	NR	NR	NR
uOmnispace.MR	K233186	510(k)	Deep Learning	MRI image visualization and navigation	NR	NR	17.5	NR	NR	NR	NR
uPMR 790	K234154	510(k)	Deep Learning	General diagnostic MRI imaging	NR	NR	32	NR	NR	NR	NR

† Device had missing or incomplete publicly available FDA summary documentation. * Ranges reflect values reported across multiple studies, datasets, or use cases in FDA summaries. ** Lower bound reported in FDA summary; no single-point estimate provided. Abbreviations: NR = Not Reported; AI = Artificial Intelligence; CNN = Convolutional Neural Network; CT = Computed Tomography; MRI = Magnetic Resonance Imaging; PET = Positron Emission Tomography; AI/AN = American Indian or Alaska Native; NH/PI = Native Hawaiian or Other Pacific Islander; Unknown = Not reported or unspecified; Other = Racial or ethnic group not otherwise categorized.

## Data Availability

The original contributions presented in this study are included in the article. Further inquiries can be directed to the corresponding author.

## Supplementary Materials

The following supporting information can be downloaded at: https://www.mdpi.com/article/10.3390/biomedicines13123005/s1, Table S1: FDA Approval Timelines by Regulatory Pathway, Country of Origin, and Clinical Panel.
